# Mitochondrial and photosynthetic therapy: A crucial strategy for remodeling cellular metabolic function

**DOI:** 10.1002/btm2.70027

**Published:** 2025-05-19

**Authors:** Muhammad Samee Mubarik, Zizhen Zhao, Mehdi Khoshnamvand, De‐Sheng Pei, Ailing Fu

**Affiliations:** ^1^ College of Pharmaceutical Science Southwest University |Chongqing China; ^2^ Caspian Sea Ecology Research Center (CSERC), Iranian Fisheries Science Research Institute (IFSRI) Agricultural Research, Education and Extension Organization (AREEO) Sari Iran; ^3^ School of Public Health Chongqing Medical University Chongqing China

**Keywords:** cellular new energy resources, chloroplasts, clinical applications, mitochondria

## Abstract

Extranuclear organelle transplantation, an emerging field in cell biology and bioengineering, presents innovative therapeutic possibilities by transferring organelles such as mitochondria between cells or across species. In living organisms, mitochondria and chloroplasts are closely related to converting substances and energy within cells. Transplantation therapy of mitochondria seeks to rebuild cell metabolic function in diseased or damaged cells and has broad application potential in treating metabolic diseases. The therapies provide a distinctive technology for cellular restoration by targeting energy generation at the organelle level, which will offer new energy resources for animal cells. At present, mitochondrial transplantation therapy has been applied as a novel approach to rescue patients in clinical settings, and chloroplast‐based transplantation endows animal cells to utilize light energy (photosynthetic animal cells). In this review, we discuss the exciting development and application prospects of mitochondrial and photosynthetic therapy in biomedicine. The technology of extranuclear transplantation would exert innovative and profound impacts on biological therapy.

**Translational Impact Statement**

Mitochondrial and photosynthetic therapy represent groundbreaking approaches to treating metabolic and hypoxic diseases by restoring cellular energy production and enhancing oxygen supply. These innovative therapies hold immense clinical promise for conditions lacking effective treatments, offering new hope for patients through the remodeling of cellular metabolism and synthetic biological advancements.

## 1 INTRODUCTION

Extranuclear organelle transplantation concerns the transfer of organelles from one cell to another or across species. Mitochondria and chloroplasts are essential organelles responsible for cellular metabolism and energy production.^1^, ^2^ According to the endosymbiotic theory, mitochondria originate from ancient alpha‐proteobacteria or other primitive bacteria,^3^ whereas chloroplasts evolve from ancient cyanobacteria.^4^ So, it is courageously speculated that the integration of mitochondria and chloroplast‐containing vehicles into the organelle network of modern cells could occur by artificial means. Because energy metabolism is necessary for all life activities, energy deficiency can lead to widespread structural and functional damage to cells.^5^ Thus, transplantation of mitochondria gains particular interest due to their potential to treat metabolic disorders by restoring normal cell function in damaged or dysfunctional cells.

Up to now, mitochondrial transplantation has been a new biotechnology in clinical^6^, ^7^, ^8^ and chloroplast‐based transplantation, while still largely experimental, explores the possibility of introducing photosynthetic capabilities into non‐photosynthetic organisms to produce oxygen and energy in hypoxic or energy‐deficient conditions.^9^, ^10^, ^11^, ^12^ By harnessing the unique function of extracellular organelles represented by mitochondria, a crucial strategy that could address disorders has been developed, particularly for diseases that currently lack effective treatments.

### 1.1 Mitochondrial transplantation therapy

Mitochondria are the primary cellular organelles responsible for producing substantial ATP.^1^, ^13^, ^14^, ^15^ In addition to their role in material and energy metabolism, they also function as a hub for intracellular signal transduction.^16^, ^17^ Mitochondria have a high sensitivity to the cellular environment, whereby numerous factors like cellular nutritional status, pH, and oxygen concentration can potentially induce transformation in mitochondrial structure.^18^, ^19^ Despite the ability of mitochondria to eliminate damaged ones and their mitochondrial DNA (mtDNA) by fusion, fission, and autophagy, external environmental changes such as hypoxia, excessive fat, and lipopolysaccharides could cause permanent injury to mitochondria as a whole.^20^, ^21^ Furthermore, genetic mitochondrial disorders produce a widespread reduction of mitochondrial function.^22^ Under such circumstances, single agents would lack the ability to stimulate mitochondrial dynamics for efficient removal or repair of the damaged mitochondria, and the mitochondrial impairment can trigger the release of excessive reactive oxygen species (ROS), cytochrome C, and other apoptosis‐related proteins, and then worsen the damage to different organelles.

### 1.2 Intracellular mitochondrial transplantation

The capacity to take up modern mitochondria is still present in cells. Isolated mitochondria could directly enter mammalian cells after simple coincubation, first observed in 1982 and subsequently identified after two decades.^23^, ^24^ Intravenous injection of mitochondria in mice allows them to be widely distributed in animal tissues and organs.^25^ While the procedure is straightforward, the internalization efficiency of intact mitochondria varies depending on the type and condition of the recipients. Increasing evidence suggests that mitochondria arrive in cells by energy‐consuming pathways such as endocytosis and micropinocytosis.^26^, ^27^ Thus, tumor cells possessing robust phagocytic capabilities exhibit high efficiency in absorbing mitochondria, whereas cells with energy deficits and less competent phagocytes often have low efficiency in mitochondrial entry.

Mitochondria undergo a differential fate upon entering cells based on the specific cell type. In most cells, external mitochondria can engage with other organelles within the cell, therefore substituting the cellular mitochondria to function.^28^ Nevertheless, a fraction of the external mitochondria enters lysosomes for breakdown in specific cells, while another fraction controls cellular activity.^29^ While there is now no definitive information about the determinants of the destiny of foreign mitochondria inside cells, it is plausible that their destiny is associated with the type and the metabolic condition of cells.

### 1.3 Mitochondrial transplantation for disease treatment

This is one of the most vibrant areas of research in mitochondrial function. Given that various diseases are associated with metabolic abnormalities and mitochondria play a crucial role in cellular metabolism, the administration of healthy mitochondria to remedy metabolic disorders would be reasonable.^30^, ^31^, ^32^ In genetic cases caused by mitochondrial deficiency, it is imperative to provide an adequate number of healthy mitochondria. Due to the diversity of mitochondrial metabolic functions, a solitary agent could not entirely substitute mitochondria or reinstate the functionality of impaired mitochondria. However, healthy mitochondrial replacement would produce a therapeutic effect that those medications are unable to achieve.

Currently, there has been significant growth in global research on mitochondrial transplantation therapy.^33^, ^34^, ^35^, ^36^, ^37^ Until now, biotechnology has been applied to transport healthy mitochondria into various cells, such as nerve, muscle, heart, kidney, liver, skin, and hematopoietic cells, to treat more than 50 abnormalities, including experimental Alzheimer's disease, Parkinson's disease, depression, schizophrenia, myasthenia, myocardial ischemia, fatty liver, rare diseases, and aging.^38^, ^39^, ^40^ Nevertheless, mitochondrial transplantation therapy continues to broaden its application in biomedicine, and a standard clinical treatment regimen was established this year.^41^

Moreover, the intracellular molecular mechanism underlying mitochondrial transplantation therapy is elucidated. After mitochondria arrive in cells, they can transform intracellular metabolites like pyruvate and amino acids to produce ATP in the cell, which is consistent with the function of mitochondria themselves.^42^ Meanwhile, the mitochondrial respiratory chain transforms NADH into NAD^+^, activates NAD^+^‐dependent proteins such as SIRT family members,^43^ and then the activated proteins promote mitochondrial‐nuclear retrograde communication and trigger the cell's protective and repair processes. Collectively, exogenous mitochondria can integrate and cooperate with the recipient cell to recover cell viability and organ function through remodeling mitochondrial metabolism and cell signaling networks.^44^

Nevertheless, mitochondria can trigger apoptosis in malignant tumor cells,^40^ which is associated with healthy mitochondria that cannot tolerate the acidic and hypoxic hostile microenvironment in tumor tissue.^44^ Unlike mitochondrial swelling caused by ischemia and hypoxia in normal tissues, tumor mitochondria display morphological changes consistent with metabolic reprogramming, such as shrinking and irregular phenotypes observed under electron microscopy. Also, the mitochondrial numbers decrease, and mtDNA mutations occur.^45^ When exogenous mitochondria are extensively taken up by tumor cells, drastic changes in the intracellular milieu may potentially trigger cell apoptosis.^46^ However, several studies show that the recruitment of mitochondria into tumor cells results in the provision of energy to the cells, promoting tumor cell growth and invasion.^47^ The reason for this opposite phenomenon may be related to the variables such as the specific kind of tumor cell, the entry number, and the quality of mitochondria.

### 1.4 Extracellular mitochondrial transplantation

Given that isolated mitochondria possess intact structure, they should have the complete function of metabolic conversion and ATP production outside cells. The uptake rate of mitochondria by diseased cells with energy deficiency is slow and inefficient, but mitochondria can still rapidly improve the survival rate and vitality of such cells.^48^ Therefore, it is speculated that mitochondria have certain noninternalized functions outside the cells. Moreover, increasing evidence shows that a certain quantity of mitochondria exists in extracellular fluid such as tissue fluid, blood, and cerebrospinal fluid,^49^, ^50^ suggesting that mitochondria might function as “secretory organelles” to exert broader regulatory effects than expected.^51^ An oral mitochondrial transplantation therapy system has been prepared,^52^ meaning that mitochondria cannot be viewed solely as an intracellular organelle, and their extracellular actions may have potentially great significance and value.

### 1.5 Clinical applications of mitochondrial transplantation

Mitochondrial transplantation therapy has not only achieved encouraging outcomes in animal models of diseases but has also shown success in several human trials. This review, based on the information from ClinicalTrials.gov, literature, and online sources, summarizes the clinical studies conducted on human mitochondrial transplantation therapy (Table 1). These clinical trials encompass mitochondrial transplantation/enhancement of hematopoietic stem cells against Pearson syndrome, autologous mitochondrial transplantation against myocardial injury and extracorporeal membrane oxygenation (ECMO) complications, autologous mitochondrial transplantation for brain ischemia treatment, and mitochondrial transplantation from heterologous UC‐MSC against refractory polymyositis and dermatomyositis and mitochondrial administration for treating chronic wounds. As an emerging clinical intervention technology, mitochondrial transplantation therapy is anticipated to shift from treating critical illnesses and rare disorders to preventing and treating common ailments.

**TABLE 1:** Clinical applications of mitochondrial transplantation therapy.

Clinical disease	Mitochondrial origin	Transplantation method	Executive agency	Reference sources
Congenital heart disease	Autologous skeletal muscle	Intracoronary injection	Boston Children's Hospital, USA	Emani^53^
Complications of extracorporeal membrane oxygenation (ECMO)	Autologous skeletal muscle	Intramyocardial injection/proximal aortic injection	Boston Children's Hospital, USA	NCT02851758^54^
Myocardial infarction/myocardial ischemia		Intracoronary and intramyocardial injection	Tehran University of Medical Sciences, Iran	NCT05669144^55^
Severe coronary atherosclerotic heart disease	Autologous platelets	Ischemic intramyocardial injection	The First Affiliated Hospital of Xi'an Jiaotong University, China	Willerson^56^
Cerebral ischemia	Autologous muscle tissue	Cerebral vascular perfusion	University of Washington, USA	NCT04998357^57^
Mitochondrial DNA deletion syndromes (Pearson syndrome, Kearns‐Sayre syndrome)	Heterologous placenta‐derived mitochondria	Autologous CD34+ hematopoietic stem cells and progenitor cells mitochondrial transplantation and reinfusion	Minovia Therapeutics, Israel	NCT06017869/NCT03384420^58^
Refractory polymyositis or dermatomyositis	Allogeneic umbilical cord mesenchymal stem cells	Intravenous injection	South Korea Paean Biotechnology Inc.	NCT04976140^59^
Chronic wound healing	Skeletal muscle	Intramuscular injection	Gulhane Training and Research Hospital, University of Health Sciences, Ankara, Turkey	Taner^60^

*Note*: The table does not include clinical trials involving the transplantation of mitochondria into germ cells.

The earliest and most widely used clinical application of mitochondrial transplantation therapy is to rescue critical patients with heart disease, including congenital cardiac disease, myocardial ischemia, and myocardial infarction.^61^, ^62^ For instance, local injection of mitochondria isolated from autologous skeletal muscle into infant cardiomyocytes treats cardiogenic shock caused by myocardial ischemia–reperfusion injury after open heart surgery.^63^, ^64^ Mitochondrial transplantation significantly increases the success rate of ECMO therapy so that 80% of patients can detach from respiratory assist machines. In comparison, the proportion is only 30% of the patients receiving standard treatment. In addition, the transplanted mitochondria improve myocardial function and reduce the incidence of major adverse cardiovascular events.^65^, ^66^

Mitochondrial transplantation therapy is also used to treat rare diseases that lack effective medical means. Pearson syndrome is a sporadic disease caused by mtDNA deficiency.^67^ Most cases die in their first year after birth due to severe bone marrow hematopoietic failure, and there are no effective ways to prevent the disease clinically. However, mitochondrial transplantation therapy allows them an opportunity to survive. The patient's mother can be the donor of healthy mitochondria. The mitochondria are transplanted into the patient's hematopoietic stem cells in vitro, and then the stem cells containing the healthy mitochondria are transfused back into the patient's body.^58^ A novel mitochondria/rAAV‐based system showed a potential approach for delivering IGF‐I to human osteoarthritic chondrocytes, effectively enhancing cell function and cartilage repair. This innovative approach holds promise as a therapeutic strategy for OA, leveraging mitochondrial transplantation for targeted gene therapy.^68^ The therapeutic potential of mitochondria transfer was adopted in treating mitochondrial diseases like Leigh syndrome. By improving lifespan, neurological function, and energy metabolism in Ndufs4−/− mice, this approach offers a promising strategy for future mitochondrial disease therapies.^69^ Clinical results show that the efficacy of mitochondrial transplantation therapy is encouraging; the unique therapeutic approach extends patients' lives and improves their quality of life.

The results obtained from animal studies and clinical applications demonstrate that animals do not exhibit any obvious adverse reactions or immune responses after mitochondrial transplantation. The body's tolerance to exogenous mitochondria is beyond what was expected. At present, clinical applications of mitochondrial transplantation are still emerging, with potential applications in treating chronic and common diseases such as anti‐aging https://longevity.technology/news/cellvie-closes-5-5m.^38^ These successful clinical outcomes will encourage further attempts to apply mitochondria in the treatment of these diseases, sparking curiosity and intrigue in the potential of mitochondrial transplantation therapy.

## 2 PHOTOSYNTHETIC THERAPY

Chloroplasts are widely distributed in green plants. Chloroplasts have a unique ability to utilize light energy to convert CO_2_ and water to glucose and oxygen (O_2_), the essential energy source for animals, meanwhile producing ATP and cellular reducing molecules like NADPH. Although mammals cannot produce O_2_, they are heavily dependent on it. Once intracellular O_2_ concentration decreases, various hypoxic diseases can occur, which is opposite to plants containing chloroplasts that can grow in hypoxic environments. Advanced animal cells need O_2_ to oxidize and decompose substances in mitochondria to generate CO_2_, while chloroplasts can convert CO_2_ into O_2_ freely. In case the characteristics of chloroplasts are combined with animal cells containing mitochondria, a new type of cell that could be called “photosynthetic animal cells” (or planimal cells) would be established. The potential of photosynthetic therapies in treating hypoxic diseases is a fascinating area of research that holds great promise. If organelle transplantation is applied to treat hypoxic diseases, the biotechnology is referred to as photosynthetic therapy.^70^, ^71^

### 2.1 Chloroplasts in animal cells

Early studies indicated that animal cells could adapt to the photosynthesis of plant chloroplasts. Some lower animals can directly take up chloroplasts and utilize the photosynthesis to survive, such as marine gastropods *Elysia chlorotica* (sea slug), *Tridachiella Diomedea*, and *Placobranchus ianthobapsus* (*Sacoglossa*, *Opisthobranchia*).^72^, ^73^ Moreover, it was observed in the early years that isolated chloroplasts could enter suspension‐cultured mouse fibroblasts (mammalian cells), and the chloroplasts maintained their integrity in the cytoplasm for at least 1 day.^74^ However, human cells do not seem to directly utilize the naked chloroplasts. After chloroplasts arrive in human cells, they will quickly rupture, and the red fluorescence of chlorophyll spreads throughout the cell.^75^ Nevertheless, exploration has been ongoing through other transplantation ways to introduce the protected chloroplast into cells due to the potential application value of photosynthesis.

### 2.2 Photosynthetic vertebrates

*Synechococcus elongatus* (*S. elongatus*) is a single‐celled autotrophic prokaryote that harbors chloroplasts. To elucidate the effect of chloroplasts on vertebrate cells, a simulation experiment of intracellular symbiosis through microinjection of *S. elongatus* into a one‐cell embryo of zebrafish is conducted.^76^ Trace observation reveals that during embryonic development, all embryonic cells contain *S. elongatus*, including the brain and eye lens, and the cyanobacterium remains viable within the embryonic cells for up to 12 days. In addition, *S. elongatus* does not have any notable effect on the morphological development of zebrafish, exhibiting good biocompatibility and biosafety.^77^ On the contrary, injection of *E. coli* or inactivated *E. coli* into zebrafish embryos would result in cell death. These findings suggest that artificially transferred *S. elongatus* can survive and undergo photosynthesis within animal cells without causing damage to the host.

A similar result is also observed from the microinjection of the microalgae *Chlamydomonas reinhardtii* into zebrafish eggs. The microalgae have the ability to spread throughout various tissues, and the symbiosis can develop into fish‐algae hybrids within the experimental period, evidenced by microscopic observation, mRNA expression in vivo, and reproduction of the algae in vitro.^78^ Moreover, the microalgae do not cause an obvious inflammatory reaction in zebrafish. The work provides supplementary evidence to establish the practicality of creating photosynthetic vertebrates.

### 2.3 Photosynthetic mammalian cells

Mammalian cells possess diverse ways to inhibit the proliferation and dissemination of exogenous bacteria.^79^ Nevertheless, the interaction between the invasion of the bacterial surface and β1 integrin of the mammalian cell membrane initiates cellular uptake of bacteria, which is a crucial step for successful bacterial invasion into host cells.^80^ Thus, bacteria that express invasion can efficiently invade mammalian cells in culture, including HeLa, U2OS, HepG2, and CHO cell lines.^70^ In addition, listeriolysin O (LLO) can disrupt endosomal membranes, allowing bacteria to be released from endosomes and enter the cytoplasm after uptake. After introducing *S. elongatus* containing invasion and LLO genes into mammalian cell culture media, the cyanobacterium can smoothly enter the cytoplasm of macrophages and CHO cells.^81^ As observed using confocal microscopy and sorted by flow cytometry, it is demonstrated that each CHO cell contains approximately one microorganism, forming an engineered photosynthetic endosymbiotic cell. Under dark conditions, *S. elongatus* engulfed by macrophages rapidly loses its red spontaneous fluorescence of chloroplasts within 12 h. However, under light exposure, red fluorescence in macrophages significantly increases within 48 h after macrophage infection with *S. elongatus*, implying that the microalgae could divide and proliferate in the mammalian cells. Meanwhile, metabolites produced and secreted by the endosymbiotic bacteria can be detected in cells. Functional studies indicate that *S. elongatus* can partially improve respiratory defects in macrophages by converting ADP to ATP, thereby supplying energy for cells exposed to dim sunlight.^9^ Biosafety analysis shows that *S. elongatus* has almost no notable effect on its host cells during the experimental period. These results suggest that *S. elongatus* can coordinate with the eukaryotic system, and two types of cells may coexist harmoniously.

Based on the symbiotic relationship between the photosynthesis of *S. elongatus* and mammalian cells,^9^, ^11^, ^12^ it is possible to artificially design photosynthetic bacteria that can survive in the cytoplasm of mammalian cells. These non‐pathogenic and non‐immunogenic photosynthetic bacteria would have the potential to serve as a promising synthetic biological carrier to endow new functions to host cells.

### 2.4 Photosynthesis enhances oxygen supply to animal brain and heart

Mammals themselves cannot produce oxygen but are highly dependent on oxygen. In particular, the activity of animal neurons requires energy provided by mitochondrial aerobic respiration, which hinges on a constant supply of external oxygen. The high dependence on oxygen has led to various neurological and psychiatric disorders that are associated with low oxygen levels in brain tissue. To enable animals to produce sufficient endogenous oxygen, single‐cell green algae and cyanobacteria containing chloroplasts were individually injected into *Xenopus laevis* tadpoles through the heart.^10^ After the determination of oxygen content in brains, it is found that these microorganisms generate a large amount of oxygen in the presence of light. Furthermore, in an oxygen‐deprived condition that can cause a complete cessation of animal neuronal activity, photosynthesis of the chloroplasts can trigger the restart and repair of neuronal activity. The results suggest that once photosynthesis is introduced into the animal body, it could directly elevate the oxygen levels in the animal brains in a controllable manner.^10^

Moreover, the heart is another organ that consumes a large amount of oxygen. The oxygen production capacity of chloroplasts may help alleviate myocardial hypoxia. After *S. elongatus* is introduced into mouse heart cells, chloroplasts in the bacteria can convert CO_2_ and water into oxygen and glucose under light. The symbiosis does not impact cell growth and normal function.^82^ When the bacteria are injected into the mouse heart that is exposed to light by surgery, oxygen generated by the bacteria is promptly taken up by the hypoxic cardiomyocytes, resulting in a substantial increase in blood supply to the heart. Additionally, the animal's immune system does not produce any rejection response to the bacteria.

Nevertheless, the production of oxygen using photosynthetic bacteria in mammalian organisms is limited by the need for sufficient and sustained light sources.^83^ However, this problem can be addressed by nano‐luminescent technology.^84^ The technology uses core–shell Nd^3+^‐doped upconversion nanoparticles (UCNPs) to convert near‐infrared light with a wavelength of 808 nm into visible light, driving *S. elongatus* to metabolize CO_2_ to O_2_. In animal models of cerebral ischemia, UCNP/*S. elongatus* could protect neuronal cells from death caused by ischemia and hypoxia.

Regarding biosafety, *S. elongatus* has good cell compatibility with mammalian cells.^85^ Compared with the control mice injected with *S. aureus*, mice receiving the same dose of *S. elongatus* do not show apparent weight loss, and the tested physiological indicators remain unchanged. Also, *S. elongatus* does not induce hemolysis, platelet aggregation, inflammation, or any other undesirable responses.

### 2.5 Application of thylakoid photosynthesis in vivo

Thylakoids are membrane structures that make up the chloroplasts and play an essential role in photosynthesis. The light reaction of photosynthesis, which captures light to produce ATP, NADPH, and oxygen, occurs at thylakoids. To ensure their stable presence within cells, the isolated thylakoids are wrapped using mammalian cell membranes to form nano‐thylakoids.^86^ Under the light, the thylakoids in mouse chondrocyte membranes can undergo photosynthesis to produce ATP and NADPH. Upon local injection of nano‐thylakoids into the cartilage‐damaged regions in an osteoarthritis mouse model,^87^ it was found that the nano‐thylakoids can rapidly enter the cells and are ubiquitously distributed throughout the cartilage tissue within 24 h of injection. As light passes through the mouse skin and reaches the interior of chondrocytes, the levels of ATP and NADPH produced by thylakoids in chondrocytes significantly increase, thereby enhancing the metabolic capacity of the cells and improving the joint health of model mice.^88^

## 3 CONCLUSION AND PROSPECT

Historically, organelle transplantation mainly indicated nucleus transfer, which will alter the genetic phenotype of organisms. However, there has been a specific emphasis on extranuclear organelle transplantation in recent years since the transplantation presents exciting opportunities for therapeutic intervention. Mitochondria and chloroplasts are the critical organelles that are responsible for cellular metabolism and energy production. According to the endosymbiosis theory, mitochondria and chloroplasts originate from ancient primitive bacteria. Now the translation of this symbiotic biological event into biomedical applications will provide new energy and resources for cells and then reshape cellular metabolic functions. Their medical values have exceeded their roles as organelles. At present, mitochondrial transplantation therapy has been employed in clinical settings, starting from critical and rare diseases to other common diseases.

Regarding the clinical application of mitochondrial therapy, a standard protocol should be established, including the source of mitochondria, mitochondrial quality control, and evaluation of therapeutic efficacy. The preferred mitochondria are autologous mitochondria of patients, followed by human stem cell‐derived mitochondria such as patient‐specific iPSC, in order to reduce the immunogenicity of mitochondria from allogeneic sources. Mitochondrial quality control is involved in the evaluation of mitochondrial membrane integrity and metabolic function, which could be achieved through biochemical assays. Consensus is needed on isolation methods (e.g., differential filtration), dosing (e.g., 2 × 106 mitochondria/gram tissue), and delivery routes (e.g., intracoronary injection). Regulatory agencies should establish guidelines for Good Manufacturing Practice (GMP)‐compliant production.

Photosynthetic therapy requires tailored protocols for light delivery, such as upconversion nanoparticles for deep‐tissue penetration, and containment strategies to prevent ecological risks. Modular biomaterial platforms (e.g., hydrogels for sustained release) could bridge gaps between lab and clinic. Global collaborations, like the MITO2i working group, are vital to harmonize research and accelerate approvals.

Emerging technologies, such as 3D bioprinting and organ‐on‐a‐chip models, can refine therapeutic efficacy and safety.^41^ For mitochondrial therapy, patient‐specific iPSC‐derived organoids enable personalized dosing and metabolic matching. Photosynthetic approaches may integrate optogenetics to control oxygen production dynamically. Combining these therapies with nanotechnology (e.g., mitochondrial‐targeted drug carriers) could enhance precision. Longitudinal studies in large‐animal models and phased clinical trials will validate long‐term benefits. By addressing these challenges through interdisciplinary innovation, mitochondrial and photosynthetic therapies can transition from experimental treatments to mainstream regenerative medicine, offering hope for metabolic and hypoxic diseases.

Mitochondrial and photosynthetic therapies hold transformative potential but face significant translational challenges. Ensuring safety requires rigorous quality control and understanding of mitochondrial and chloroplast behavior post‐transplantation. Immune compatibility demands innovative engineering to evade host defenses, while standardized protocols and regulatory guidelines are essential for clinical adoption. Collaborative efforts among researchers, clinicians, and regulators will be critical to advancing these therapies from bench to bedside. The exploration of chloroplast‐based transplantation will fundamentally create artificial animal chloroplasts. The development and exploitation of biotechnology that inputs new energy sources for mammalian cells holds great potential to revolutionize regenerative medicine and synthetic biology, offering a new paradigm for treating a wide range of medical conditions.

## AUTHOR CONTRIBUTIONS

Muhammad Samee Mubarik and Zizhen Zhao wrote the draft. Mehdi Khoshnamvand revised the draft. De‐Sheng Pei and Ailing Fu proposed the conception.

## FUNDING INFORMATION

This work was supported by Chongqing Technology Innovation and Application Development Sichuan‐Chongqing Special Key Project (CSTB2024TIAD‐CYKJCXX0017), High‐level Talents Project of Chongqing Medical University (No. R4014) and the Chongqing Postdoctoral Innovation Mentor Studio (X7928 D.S.P.).

## CONFLICT OF INTEREST STATEMENT

The authors declare no conflicts of interests.

## Data Availability

The data that support the findings of this study are available from the corresponding author upon reasonable request.
